# Hydraulic fracturing flowback chemical composition diversity as a factor determining possibilities of its management

**DOI:** 10.1007/s11356-021-16432-7

**Published:** 2021-10-13

**Authors:** Joanna Fajfer, Olga Lipińska, Monika Konieczyńska

**Affiliations:** Polish Geological Institute – National Research Institute, Rakowiecka 4, 00-975 Warsaw, Poland

**Keywords:** Flowback fluid, Hydraulic fracturing, Management of flowback fluid

## Abstract

The chemical characteristic of flowback fluid from hydraulic fracturing for shale gas exploration/production in various localizations is presented. The results of statistical analysis have shown that variability in the chemical composition of these fluids is statistically significant and depends on the time difference between fracturing process and flowback sampling as well as sampling spot within the installation for flowback collection. Parameters which depend on sampling schedule (time and spot of sampling) are as follows: electrical conductivity and concentration of ammonia, boron, barium, calcium, lithium, sodium, magnesium, manganese, sodium, strontium, silicate, bromide, and chloride. Independent parameters are pH, total organic carbon (TOC), concentration of potassium, and iron. The ranges of the values of the characteristic parameters were determined, taking into account the representativeness of the samples, supported by statistical tests. The methods for the reuse of flowback fluids in terms of chemical composition are presented.

## Introduction

The common method used for exploration and exploitation of unconventional hydrocarbons (tight gas, shale gas, tight oil) is hydraulic fracturing of the reservoir rock through horizontal borehole. Hydraulic fracturing increases hydrocarbon inflow from reservoir rock by making small fractures and increasing reservoir permeability (Fajfer et al. 2016; Oetjen et al. 2017). The method rests on pumping a large amount of fracturing fluid into the reservoir. A high pump rate (6–20 m^3^/min) allows achieving high pressure up to 100 MPa on the inlet to the well (Steliga and Uliasz 2012; Pakulska 2015). After the fracturing process, part of the fluid returns from the well to the surface as a so-called flowback. Fracturing fluid is a water solution of various chemicals with a weight concentration from 0.5 to 2.5% (Fajfer et al. 2016; Pakulska 2015; Krogulec and Sawicka 2012; U.S. EPA 2016). The composition of those chemicals is adjusted to the physicochemical parameters of the reservoir. During the fracturing process, the composition of the fracturing fluid is modified according to the process stage. Physicochemical parameters of fluid flowing back to the surface depend on parameters of fracturing fluids used during the process, physicochemical properties of reservoir rocks, and formation water. Additionally, the parameters of flowback fluid depend on duration time and temperature of reaction between fracturing fluid and reservoir rock. The amount of flowback fluid depends on pressures of fluid in the reservoir, amount of reservoir water, and collection time. The flowback fluid is collected during production tests lasting several weeks. After a production well head is installed in the production phase, all fluids that come up together with hydrocarbons are called produced water, though their characteristics can still remain very similar to former flowback and they still need to be managed in a similar way.

The fracturing process consumes a large amount of water. Data collected in different countries and at different stages of exploration/exploitation presents the amount of water per one well that varies between 7500 and 38,000 m^3^ in the USA (Kuwayama et al. 2015; Vengosh et al. 2014), 2000 and 77,000 m^3^ in Canada (Jacobsen and Gravesen 2016), 10,000 and 30,000 m^3^ in the UK (Hammond et al. 2015; Stumford and Azapagic 2014), and 12,095 m^3^ in Germany (Olsson et al. 2013; Bergman 2014). In Poland, where hydraulic fracturing was performed only for the hydrocarbon exploration stage, the amount of used water varied between 1284 and 37,849 m^3^. After fracturing job stops, flowback fluid flows back through open valve on the head of a borehole (Konieczyńska et al. 2015). A large portion of the fluid is collected in the first days after fracturing which is a result of pressure equilibration in reservoir (U.S. EPA 2016). However, during the further exploitation stage, it keeps flowing back together with reservoir water as so-called produced water. It is reported that in the USA, the amount of recovered fluid varied between 10 and 80% of fluid pumped into a borehole (U.S. EPA 2016; Harrison et al. 2014; Olmstead et al. 2013; Haluszczak et al. 2014). In Europe, flowback fluid was collected only during reservoir tests in the exploration stage as so far no production from unconventional reservoirs has been started. The amount of collected fluid in Germany varied between 10 and 30% of fluid used for fracturing. In Poland, the amount varied between 16 and 30% (Konieczyńska et al. 2015). The flowback fluid contains a lot of chemicals that can pose a significant threat to the environment. Therefore, it is recommended to reuse this fluid for the preparation of another portion of fracturing fluid. However, if there is no possibility to reuse the flowback, it should be treated as waste. The appropriate treatment processes are needed in order to reach the required physicochemical parameters for flowback fluid reuse or its safe disposal.

The aim of the study is to find physicochemical parameters of flowback fluid that are crucial for the development and application of appropriate treatment processes. Because the chemical composition of flowback fluid changes along recovery time, the treatment process should be independent of variability of the physicochemical parameters and should be effective in a broad range of their values. The study was performed based on chemical and physical analyses of flowback fluids from the USA, Canada, Germany, and the UK as well as from Poland. The chemical and physical analyses of flowback fluids in Poland were performed by the Polish Geological Institute–National Research Institute (PGI–NRI) between 2010 and 2016. The statistical methods were applied for the comparison of the results of the analysis. The discussion on the results was presented in the light of waste management.

### Geological settings

The Americans have the greatest experience in the field of exploration and exploitation of unconventional hydrocarbons, where natural gas and crude oil from shale formations have been extracted since the early 90s (Curtis 2002). In Canada, exploration and exploitation of shale gas and oil started in 2005, from Montney Formation, and in 2007, from the Horn River formation (Rivard et al. 2014). However, the highest rate of development is observed in China, where the exploration started in 2005, the first exploitation tests were performed in 2008, and the first concessions for unconventional hydrocarbons production were issued in 2009 (Zhou et al. 2016). In Europe, only exploration tests have been performed, mainly in Poland where 72 localizations were tested (Ministry of the Environment 2016). Several tests were performed in Germany (Olsson et al. 2013; Bergman et al. 2014) and the UK (Pripch et al. 2015; Hudson et al. 2016). In Poland, the reservoir tests indicated low effectiveness of hydrocarbons recovery, and further exploration of unconventional hydrocarbons was ceased. In the UK, fracturing of reservoirs induced seismic activities, and thus, further exploration involving hydraulic fracturing was banned (Hammond et al. 2015).

Shale gas formations differ from each other by geological conditions, depth and thickness, organic carbon and clay content, thermal maturity, and so on. Therefore, the chemical composition and amount of fracturing fluid pumped into reservoir in each borehole are tailored to local geological conditions.

In the USA, the prospective shale gas and oil formations are located in, Texas, Oklahoma, Arkansas, and Pennsylvania (Curtis 2002; Jarvie 2012).

Those locations encompass the following geological formations: Haynesville (Upper Jurassic), Barnett (Lower Carboniferous), Marcellus (Middle Devonian), Fayetteville (Lower Carboniferous), Eagle Ford (Upper Cretaceous), and Woodford (Devonian). The depth of the reservoir depends on the location — the shallowest is Marcellus — the upper boundary is located between 1200 and 2,400 m below the surface, whereas the deepest is Haynesville formation — with the upper boundary between 3200 and 4100 m. The average thickness of reservoirs is around 45 m for Woodford and Marcellus and up to 200 m in the case of Barnett. Average organic matter content for shale formations varies between 1.3 and 5%, whereas thermal maturity from 1.2 to 1.6% Ro (vitrinite reflectance scale) (Poprawa 2010a; Janas and Dyrka 2013).

Formations with shale gas potential in Canada are located mainly in two provinces: British Columbia (upper Devonian Horn River and Muskwa formations, and lower Triassic formation Montney) and Alberta (upper Devonian Duvernay and lower Triassic Montney formations). The top of upper Devonian formations in British Columbia is located up to 2500 m below surface, and their thickness varies between 200 and 500 m. The organic matter content is up to 6%. The top of the Montney Formation lies between 500 and 4000 m of depth, with its maximum thickness of 300–400 m and organic matter content between 0.1 and 3.6% (the average 0.8%). The Muskwa Shale deposition in northwest Alberta is the northern continuation of the Duvernay Shale in central Alberta. Its top is located at 1000 m below the surface in the Eastern part and reaches 5500 m in the Western part. The thickness varies between 30 and 120 m and organic matter content between 0.1 and 11.1% (Rivard et al. 2014).

The Sichuan and the Tarim basins are reservoirs with the biggest shale gas potential in China (Changbo and Pieńkowski 2015). They are located deeper than in the USA and have a more complex geological structure (Changbo and Pieńkowski 2015). In the Sichuan basin, the gas exploitation is conducted in four regions of the Longmaxi shale formation: Fuling, Changning, Weiyuan, and Pengshui. The thickness of the formation varies between 100 and 120 m; however, the layers rich in gas are located at the bottom of the formation, and their thickness is around 30 m. The top of the formation is located between 2000 (in the Pengshui part) and 4500 m (Yang 2019). Organic matter content is above 2% and thermal maturity above 2.2% Ro (Changbo and Pieńkowski 2015).

In Germany, Lower Jurassic Posidonia Shale with 8% content of organic matter is considered as most prospective shale gas formation. It is a part of the Lower Saxonian Basin located 3000 m below the surface. The thickness of the formation varies between 15 and 30 m (Janas and Dyrka 2013).

In the UK, there are three shale formations with shale gas potential: two Carboniferous — Bowland in Northern England and Midland Valley in Scotland (Monaghan 2014), and the Jurassic one — Weald in southeastern England (Stephenson 2015; Hammond et al. 2015). The content of organic matter in Bowland formation varies between 2 and 5%, and its thermal maturity of 1.2% Ro is higher than the thermal maturity of shale from Weald formation which is only 0.8% Ro (Janas and Dyrka 2013).

In Poland, unconventional hydrocarbons are expected in fine-grain deposits of lower Paleozoic (upper Ordovician and lower Silurian shales) located in the Baltic and the Lublin Basins. In the Baltic Basin, the deposits are located between 1000 m below the surface in the Eastern part and 4500 m below the surface in the Western part (Poprawa 2010b; Karcz et al. 2013). Those formations are less permeable than conventional hydrocarbons deposits (Dyrka 2013) with thermal maturity which varies from 0.5 to 4% Ro in the Baltic Basin and from 0.6 to2% Ro in the Lublin Basin. In the Baltic Basin, deposits contain from less than 1 to 2.5% of organic matter, whereas in the Lublin Basin from less than 1 to 3% (Poprawa 2010b).

### Chemical characteristic of flowback fluids from worldwide locations

According to literature data, in the USA, the amount of flowback fluid and produced water from one borehole is in the range from 10 to 80% of fracturing fluid volume injected during the hydraulic fracturing process (U.S. EPA 2016; Harrison et al. 2014; Olmstead 2013; Haluszczak et al. 2013). In the first month, the average amount of fluid that returns to the surface is in the range of 8 to 10% (Olsson et al. 2013). After 6 months, the flowback volume is in the range from 20 to 50% (Kondash et al. 2017). In Canada, the amount of flowback fluid that returns after the fracturing process is in the range from 10 to 30% of injected volume (Bustin et al. 2018), whereas in the Sichuan Basin in China, the amount is in the range from 10 to 60% (Yang et al. 2019). In the case of Europe, hydraulic fracturing was performed only for exploration purposes; thus, produced water was not collected. In Poland, the amount of the collected flowback was in the range from 16 to 30% of injected fluid volume (Konieczyńska et al. 2015; Fajfer et al. 2016), whereas in Germany, this amount was around 25% (Olsson et al. 2013).

The scope of physicochemical analysis of flowback fluid reported in the literature is very diverse (He et al. 2013; Hayes 2012; Olsson et al. 2013; Yang 2019; Konieczyńska et al. 2011; Konieczyńska et al. 2015; Owen 2017; Owen and Bustin 2017; Liang et al. 2018; Busby 2018; EA 2011; Zolfaghari et al. 2015). Total dissolved solids concentration (TDS), pH, concentration of sodium, potassium, calcium, barium, strontium, iron, chloride, bromide, and sulfate are the most frequently reported in the literature. The data on electrochemical conductivity and total organic carbon content (TOC) are less common. Biological oxygen demand (BOD) and chemical oxygen demand (COD) are presented occasionally. Among the remaining parameters, the concentration of lead, chromium, zinc, and nickel was most often determined.

**Fig. 1:**
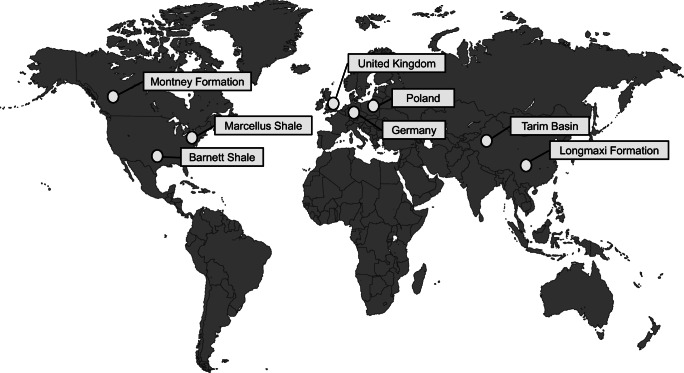
Worldwide locations of shale gas sites with available chemical characteristics of flowback fluids

The biggest dataset of chemical analysis results reported in the literature is related to flowback fluids from Marcellus and Barnett deposits (Fig. 1). Despite different chemical compositions, it has been found, on the basis of nineteen samples from Marcellus and five samples from Barnett, that the main constituent is chloride (Hayes 2012). Flowback fluids were characterized by pH within range from 4.9 to 7.9 and TDS from 680 to 261,000 mg/l (increasing with time of recovery). The hardness of the fluid varied between 630 and 95,000 mgCaCO_3_/l, whereas COD and BOD from 228 to 1900 mgO_2_/l and from 2.8 to 2070 mgO_2_/l, respectively. The concentration of chloride and sulfate was within range from 64.2 to 181,000 mg/l and from 10.3 to 348 mg/l, respectively. The concentration of sulfate was dropping down with an increase of collected flowback fluid. The concentration of bromide ranged from 35.5 to 1600 mg/l. Concentrations of other constituents were as follows: sodium from 63.8 to 75,800 mg/l, calcium from 35.2 to 24,000 mg/l, potassium from 2.69 to 3950 mg/l, barium from 0.332 to 4220 mg/l, strontium from 0.58 to 8020 mg/l, and iron from 2.68 to 158 mg/l(Hayes 2012). In case of flowback fluids from Barnett deposit, pH value ranged from 6.5 to 8.0, TDS from 5850 to 97,800 mg/l, hardness from 840 to 21,000 mgCaCO_3_/l, COD from 850 to 4280 mgO_2_/l, BOD from 89 to 2120 mgO2/l, and TOC from 6.2 to 99.1 mg/l. Concentration of chloride was within range from 3300 to 60,800 mg/l, sulfate from 120 to 1260 mg/l, bromide from 34.3 to 798 mg/l, sodium from 278 to 28,200 mg/l, calcium from 13 to 6730 mg/l, potassium from 4 to 750 mg/l, barium from 0.053 to 17.9 mg/l, strontium up to 1550 mg/l, and iron up to 93.8 mg/l. Other elements were present in fluids from both deposits at trace level (Hayes 2012).

In Canada, the flowback fluids from thirty-one boreholes in Montney Formation were analyzed (Owen et al. 2017, Bustin et al. 2018)(Fig. 1). The electrical conductivity of the fluids ranged from 15 to 200 mS/cm and pH from 2.3 to 9.5. TDS was within range from 3609 to 228,259 mg/l; however, an increase of TDS along the time of fluid recovery was observed. Similar to the USA boreholes, the main constituent in flowback fluid from Canadian boreholes was chloride, with concentrations from 1893 to 164,018 mg/l. Concentration of sulfate ranged from 0.1 to 1084 mg/l, sodium from 1344 to 51,027 mg/l, calcium from 13 to 11,705 mg/l, potassium from 49 to 1920 mg/l, magnesium from 9 to 1329 mg/l, barium from 1 to 467 mg/l, strontium from 4 to 1219 mg/l, and lithium from 1 to 49 mg/l(Owen 2017).

Chinese studies concerned flowback fluids from boreholes in the Longmaxi shale formations of the Sichuan Basin (Yang et al. 2019) and from the Tarim Basin (Liang et al. 2018)(Fig. 1). The Sichuan Basin flowback fluids were collected from six boreholes. Measured TDS ranged from 13,000 to 55,000 mg/l and pH value from 6.84 to 7.48. Concentration of other constituents varied as follows: chloride from 13,110 to 36,470 mg/l, bicarbonate from 310 to 1263 mg/l, bromide from 180 to 470 mg/l, sulfate from 41 to 172 mg/l, sodium from 8296 to 19,830 mg/l, calcium from 564 to 3870 mg/l, potassium from 157 to 768 mg/l, barium from 138 to 412 mg/l, magnesium from 105 to 336 mg/l, and strontium from 67 to 320 mg/l. Concentrations of ammonia, aluminum, and iron were at trace level, or those species were not detected in samples of investigated flowback fluids (Yang et al. 2019). In the Tarim Basin, samples of flowback fluid were collected from one borehole, and only fluid density, pH, and concentration of chloride were tested. Density ranged from 1.088 to 1.22 g/cm^3^, pH was determined between 5.86 and 6.71, and chloride concentration varied between 32,000 and 64,000 mg/l; but the maximum was measured after 6 days of recovery, and it decreased in the next days (Liang et al. 2018).

In Germany, the flowback fluid from the Dahmn-3 borehole was tested (Fig. 1). Similar to fluids from boreholes in Canada and the USA, the highest concentration was observed for chloride from 40,360 to 88,440 mg/l. Other constituents were present at lower concentrations: sodium from 17,690 to 36,390 mg/l, calcium from 6700 to 16,550 mg/l, strontium from 790 to 1720 mg/l, barium from 180 to 593 mg/l, magnesium from 890 to 2130 mg/l, potassium from 52 to 570 mg/l, and iron from 23 to 160 mg/l. Other metals were present at trace concentrations. The concentration of sulfate ranged between 4 and 15 mg/l. Data related to pH value, TDS, and TOC were not reported (Olsson et al. 2013).

In the case of the UK, the chemical analyses of twenty-four parameters were performed for fracturing fluid from Prees Hall 1 borehole located in the Lancashire County (Fig. 1). The pH value was determined within the range from 5.40 to 6.10. TDS ranged from 94,000 to 210,000 mg/l and alkalinity from 41 to 133 mgHCO_3_/l. Total suspended solids (TSS) were within the range from 23 to 2600 mg/l and COD from 120 to 3240 mg/l. Similar to flowback fluids from other locations, the highest concentration, within the range of 48,000–100,000 mg/l, was reported for chloride. The concentrations of other constituents were as follows: barium from 9.2 to 30 mg/l, iron from 4.2 to 23 mg/l, manganese from 1.60 to 2.80 mg/l, arsenic from 0.48 to 1.40 mg/l, and nickel from 0.16 to 0.88 mg/l(Busby 2018). Based on the Environment Agency (EA 2011), the concentration of potassium ranged from 28.8 to 40.6 mg/l and sodium from 9300 to 34,800 mg/l, and electrical conductivity was determined within the range from 133.73 to 176 mS/cm.

### Chemical characteristic of flowback fluids from Poland

In shale gas formations in Poland, 72 boreholes were drilled, and there were 34 full-scale hydraulic fracturing treatments performed as a part of shale gas exploration activities undertaken by the industry between 2010 and 2016 (Ministry of the Environment 2016). The PGI–NRI conducted several research projects related to the environmental impact of shale gas exploration, where the samples of fracturing fluid and flowback were collected (Konieczyńska et al. 2011; Konieczyńska et al. 2015; Kantor et al. 2015; Fajfer et al. 2016; Konieczyńska et al. 2016 unpublished results). Analytical tests were performed on samples of fracturing fluid and flowback. The samples of fracturing fluid were obtained from one borehole and represent one full-scale hydraulic fracturing treatment. The samples of flowback were collected from 8 boreholes where hydraulic fracturing was conducted (Gapowo, Lublewo, Lubocino, Łebień, Stare Miasto, Syczyn, Wysin, Zawada) in 7 of them in directional sections.

Analyzed flowback samples differed in terms of the time of collecting (counting in days after the hydraulic fracturing treatment) and sampling spot (different outlets of technological line applied on particular well pads to collect the flowback). The geographical location of the drilling and the time duration between hydraulic fracturing and collection of the samples may be the factors of differentiation of the composition of flowback and affect waste management decisions (Balashov et al. 2015; Zambrano et al. 2016; Vieth-Hillebrand et al. 2017). In addition, the differences in the results between samples taken from different spots of the same installation, e.g., upstream or downstream of the gas separator, showed that the initial process of fluid treatment takes place already within the installation, and its efficiency influences the composition of generated fluids to be managed ultimately.

## Materials and methods

In this article, the analysis of the dataset is performed to provide a characteristic of flowback fluids in Poland. For safety reasons, samples of fracturing fluid were provided by a hydraulic fracturing operator. Samples of flowback fluid were collected by trained employees according to the defined scheme for each borehole. The collected samples were tested in order to find out the chemical composition of the fluids and the variability of this composition over time, in order to assess potential threats to the environment and to identify appropriate methods of fluid management including the final disposal procedure.

Flowback sampling campaigns were each time tailored to the technical aspects of the stimulation process applied on the well pad by its industrial operator, in particular the number of hydraulic fracturing stages, the composition of fracturing fluid, and construction of the well-pad infrastructure (technological flowback recovery line). There are several outlets within the technological flowback recovery line possible to be used to collect a sample of flowback, depending on the purpose of the study. The samples collected on choke manifold represent fluid not affected by the surface operation and as the best approximation of downhole fluids may serve in studies focused on fluid–rock reaction in reservoir. Otherwise, the samples collected before and after the removal of volatile constituents indicate the actual efficacy of the separator. The samples collected from collecting tanks represent the average composition of the portion of flowback intended for management (reuse, disposal, etc.). Depending on the purpose of the research, also, the number of samples taken is different for different locations.

In total, forty samples of flowback fluid and two samples of fracturing fluids were analyzed by the Chemical Laboratory of the PGI–NRI between 2010 and 2016, within several research projects. The following parameters were analyzed: pH, electrical conductivity, turbidity, TOC, alkalinity, COD, phenol index, concentration of surface-active agents, cyanide, ammonia, bicarbonate, fluoride, chloride, nitrite, nitrate, phosphate, sulfate, aluminum, silver, arsenic, beryl, cadmium, cobalt, copper, lithium, molybdenum, nickel, lead, rubidium, antimony, selenium, tin, tellurium, uranium, vanadium, boron, barium, calcium, chromium, iron, potassium, magnesium, manganese, sodium, silica, strontium, titanium, zinc, and mercury. The samples with high salinity were diluted prior to analysis. Based on elemental analysis, TDS and hardness were computed. All analyses were performed according to Laboratory’s methods (description available at www.pgi.gov.pl). All analytical methods were validated in accordance with ISO 17025 method (accreditation certificate number AB 283, issued by Polish Centre for Accreditation). For individual samples, the set of analyzed parameters varied, as the research was carried out to facilitate various projects. The goal of the presented result analyses is to deliver a set of characteristics for flowback fluids generated in Poland.

The analyses include data verification, expert assessment of sample origin, cleaning and standardization of data, and selection and application of most suitable statistical methods. The characterization means a range of values (minimum–maximum) and medium value (arithmetical average and median) for analyzed parameters in flowback fluid. The characterization is based on selected samples, assessed as a representative, due to the high variety in dataset. The dataset is a collection of results from different research projects conducted by PGI–NRI in recent years. Parameters obtained for the samples of flowback are presented, including diversity among different locations. Also, not detected substances are revealed. Next, the comparison between fracturing fluid and flowback is given as well as time-related differentiation.

## Results

**Fig. 2:**
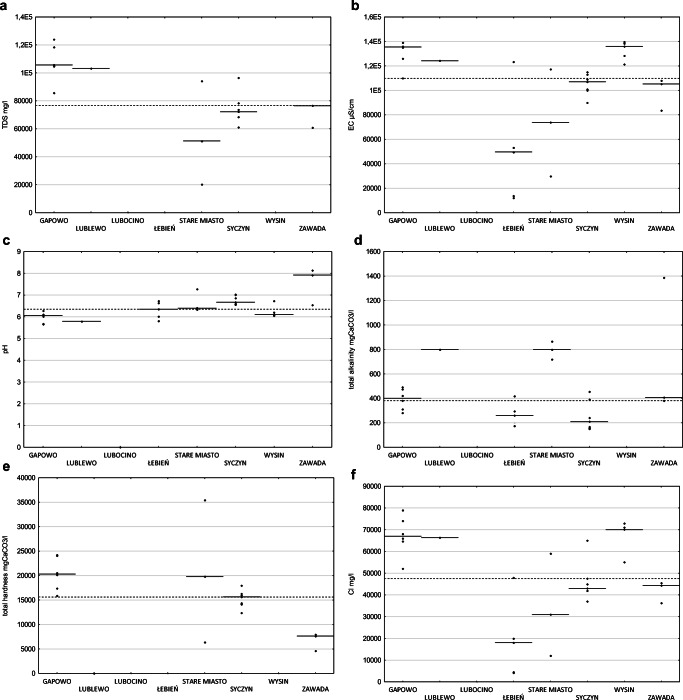

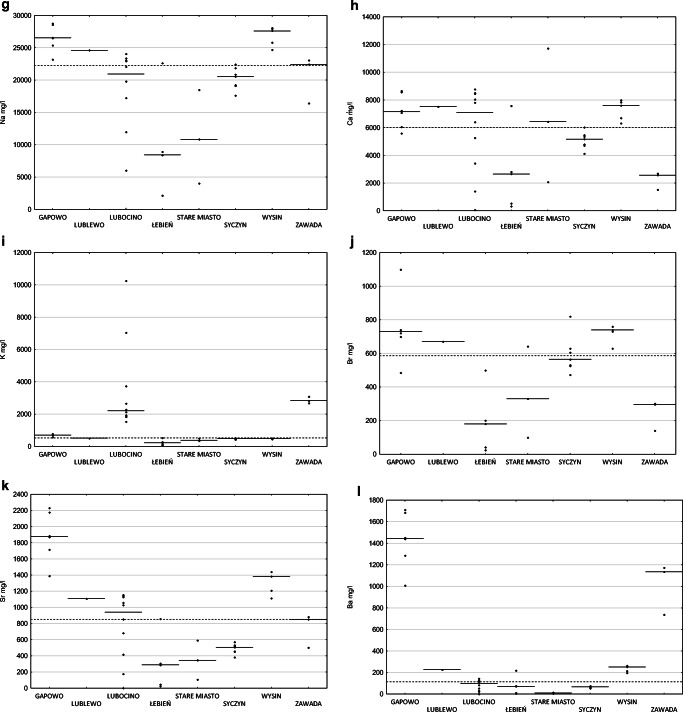

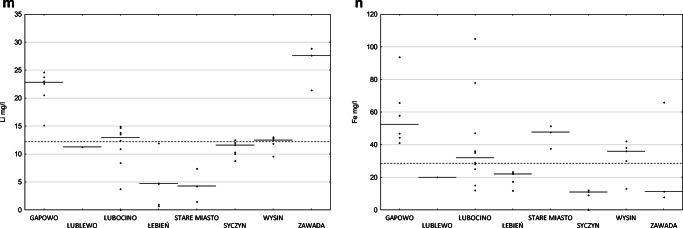
Comparison of the median values of total dissolved substances concentration (**a**), electrical conductivity (**b**), pH (**c**), total alkalinity (**d**), hardness as CaCO3 (**e**) and concentrations of chlorides (**f**), sodium (**g**), calcium (**h**), potassium (**i**), bromides (**j**), strontium (**k**), barium (**l**), lithium (**m**), and iron (**n**) for 8 sampling locations (dashed lines) and the general median for the whole set (solid line); raw data are included (diamonds). On the *x*-axis, localization of samples collection

High content of total dissolved substances was found in the tested samples of flowback (Fig. 2). TDS varied between 20,182 and 123,815 mg/l, arithmetic average 82,270 mg/l, and median 77,424 mg/l (based on 20 samples). As a result of high TDS, the high electrical conductivity of flowback was also measured. The arithmetic average of electrical conductivity measured in thirty samples was 102,577 μS/cm and median 111,500 μS/cm. For 2 out of 8 locations, the conductivity levels are low, with a minimum of 12,140 μS/cm, while for others, values over 80,000 μS/cm were more characteristic, with the maximum reaching 139,800 μS/cm (Fig. 2).

Measured pH values were from 5.66 up to 8.14 with values significantly above 7 recorded for two locations. The median pH value was 6.38, and the mean was 6.48 (based on 30 samples).

Alkalinity measured in 25 samples was in the range from 152 to 1385 mgCaCO_3_/l. Hardness measured in 17 samples ranged from 4577 to 35,418 mgCaCO_3_/l.

The main constituent of flowback was chloride; its concentration was measured in the range from 4100 to 79,000 mg/l, with an arithmetic average equal to 48,440 mg/l and median equal to 47,750 mg/l. The average of sodium concentration was 19,232 mg/l and median was 22,229 mg/l, ranging from 215 to 28,750 mg/l. Also, a significant amount of calcium seemed to be characteristic. On average, it was present in the amount of 5543 mg/l (median 6036), from 62 to 11,712 mg/l. Potassium ranged from 82 to 10,240 mg/l, on average 1422 mg/l, while the median was 529 mg/l; two samples showed extremely high (compared to the rest of the samples) potassium levels (Fig. 2).

The arithmetic average of bromide concentration was 534 mg/l, the median was 585 mg/l, and the lowest and highest concentrations were 25 mg/l and 1100 mg/l, respectively. Strontium was determined in 39 out of 40 tested samples; the highest concentration was 2230 mg/l, and the average was 870 mg/l; similarly, the median was 851 mg/l (the limit of quantitation (LoQ) is 0.3 mg/l).

The concentration of barium was in the range from 0.2 to 1710 mg/l; the average concentration was 368 mg/l, while median 115 mg/l—this significant difference is related to outliers within the dataset.

Lithium was determined in 39 out of 40 tested samples, the highest concentration was 28.9 mg/l, the average was 12.5mg/l, and similarly, the median was 12.2 mg/l (the limit of quantitation (LoQ) is 0.2 mg/l).

Concentrations of iron ranged from 7.69 to 105 mg/l. The mean iron concentration was 33 mg/l, while the median was 28.5 mg/l. In one sample, no iron was found above the quantification limit of 1 mg/l. The non-iron sample represented fluid after pretreatment; in the analogous samples representing the fluid part before the pretreatment line, the level of iron’s concentrations was 11–12 mg/l.

Moreover, in the samples of flowback fluids, the presence of silica, boron, magnesium, and manganese as well as sulfate, nitrate, and nitrite was established. Concentrations of other elements were at trace level or below the quantification limit.

**Fig. 3:**
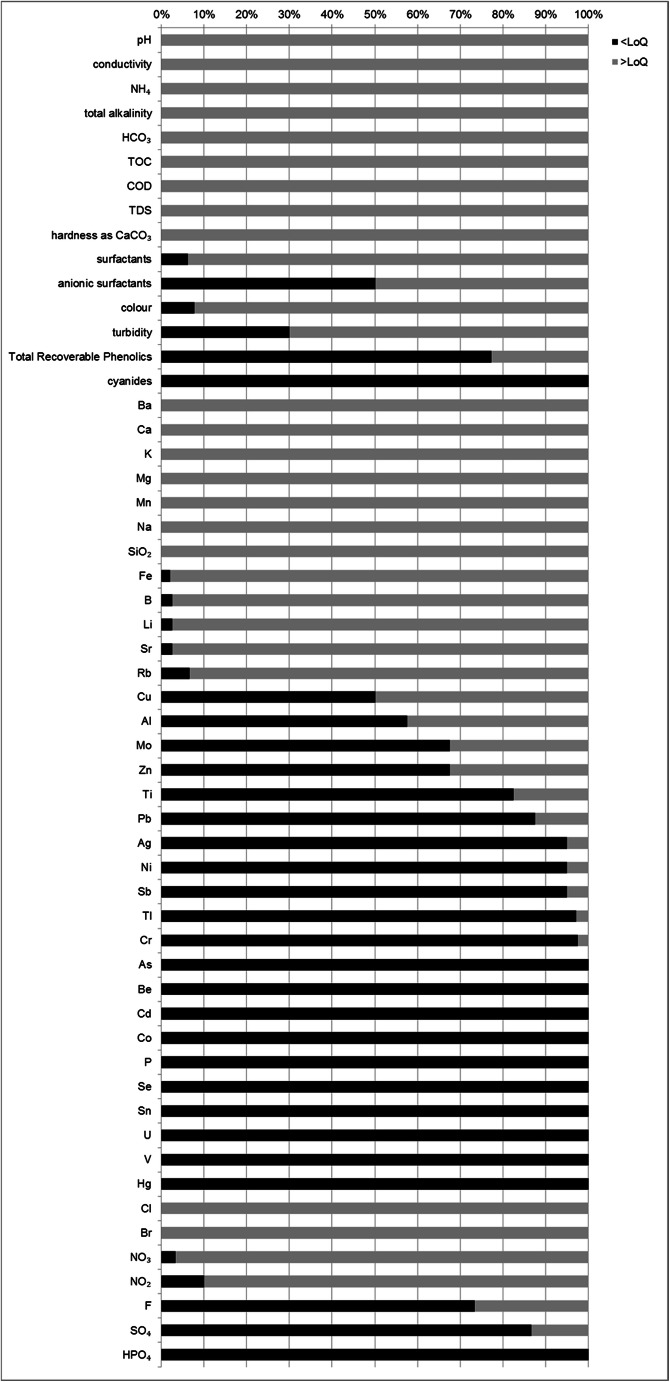
The share of parameters detected in flowback samples

There was no evidence of arsenic, beryllium, cadmium, cobalt, phosphorus, selenium, tin, uranium, vanadium, mercury, phosphates, and cyanides in any of the tested samples, whereas components such as lead, silver, nickel, antimony, thallium, chromium, fluorides, sulfates, and phenols were determined only in a few samples, at very low levels of quantification (note that in some cases, the LoQs were higher, due to high salinity of samples with a complicated matrix and need for dilution). For all analyzed parameters, the percentage share of results obtained below the LoQ of a given analytical method is presented on the diagram (Fig. 3).

**Fig. 4:**
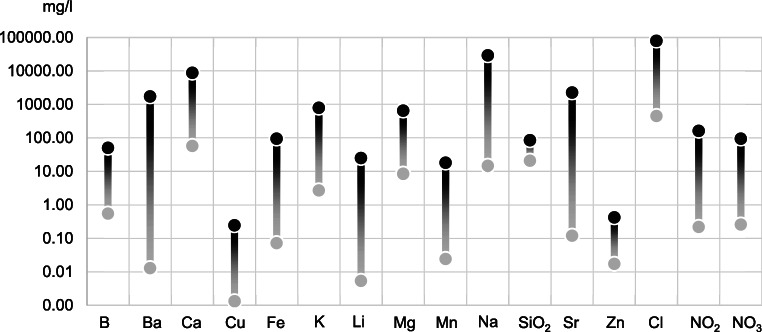
Comparison of the maximum concentrations of selected parameters in fracturing fluid (gray dots) and in flowback fluid from the same borehole (black dots); the scale is logarithmic

The comparison of the properties of the fracturing fluid and the flowback fluid clearly shows that the composition of the original fluid has been modified, most likely as a result of contact with the formation and/or reactions occurring in the fluid during and after the hydraulic fracturing treatment. The greatest increases in maximum concentrations were observed for barium, strontium, lithium, sodium, and iron (Fig. 4).

**Fig. 5:**
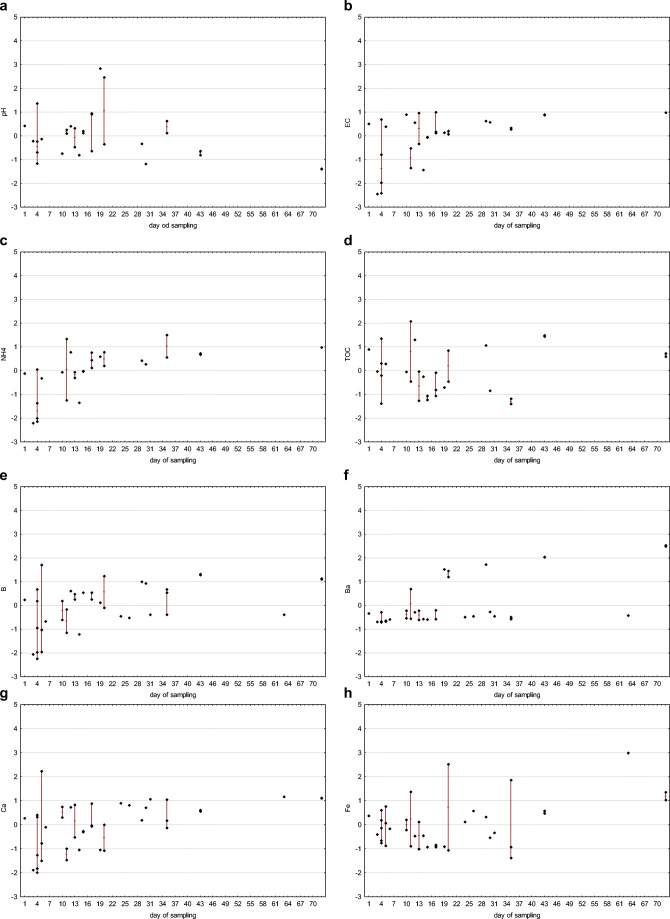

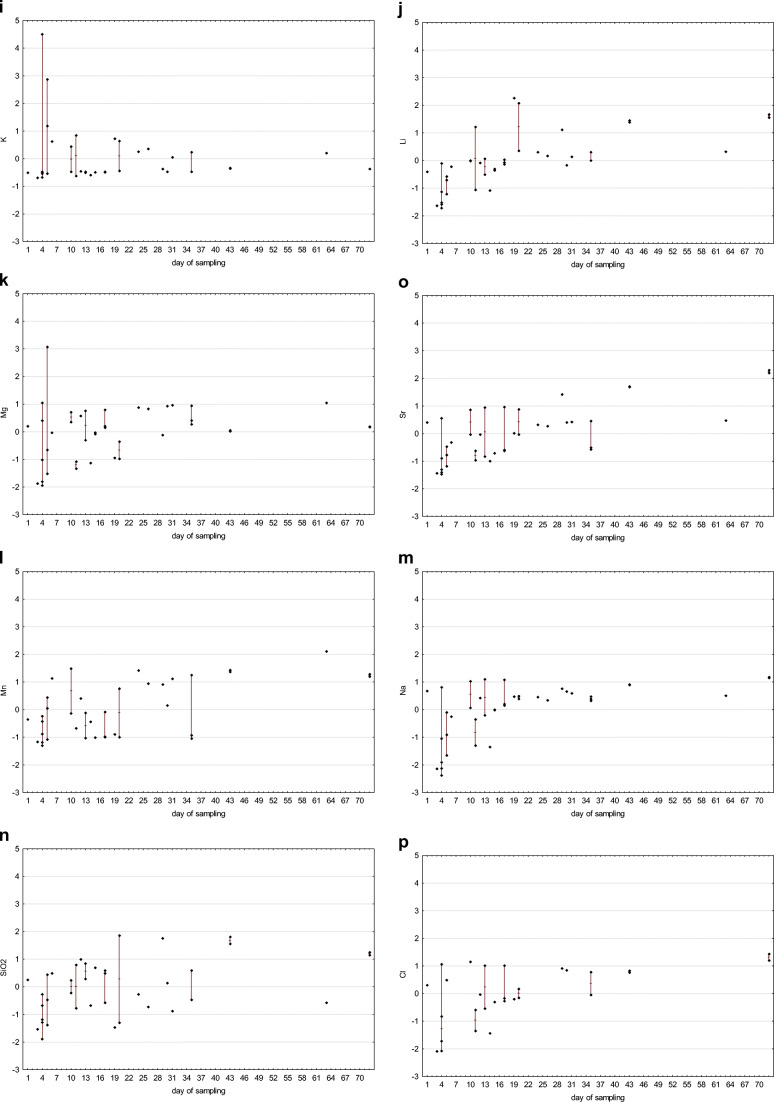

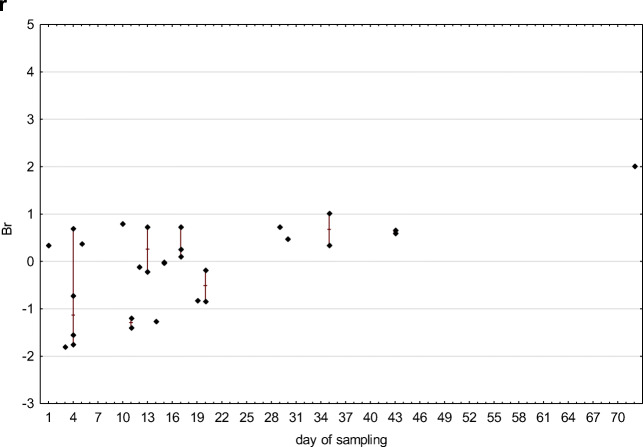
Time-related differentiation of flowback: standardized values of pH (**a**), electric conductivity EC (**b**), ammonia (**c**), total organic carbon (**d**), boron (**e**), barium (**f**), calcium (**g**), iron (**h**), potassium (**i**), lithium (**j**), magnesium (**k**), manganese (**l**), sodium (**m**), silica (**n**), strontium (**o**), chlorides (**p**), and bromides (**r**) are given (diamonds) in relation to the day of sampling (*x*-axis), maximum–minimum range is marked with a line, where appropriate

In the case of flowback fluids sampling, it should be clarified that each subsequent sample represents a portion of the fluid that may have undergone other processes than the previous one. The later the sample is collected, the more likely the original chemical composition will be transformed due to reactions taking place in the fluid itself and between the fluid and the reservoir. The longer the period, the greater opportunities to achieve equilibrium under high temperature and pressure conditions. The variability of individual parameters depending on the sampling date, taking into account standardized raw data, is presented in the charts (Fig. 5).

### Statistical analysis of fluid differentiation

The collected samples of flowback fluids, as mentioned earlier, differed in terms of the time of sampling and the place of collection. One of the methods of assessing the diversity of the analyzed variables is statistical tests, which can be used to assess whether the relationships between the variables observed in the analyzed dataset are statistically significant, which allows drawing conclusions in the general context (population). The selection of the appropriate test depends on several factors; the most important of which are the nature of the variable, sample size, and distribution.

Here, nonparametric methods were used due to the fact that the independent variables are ordinal. Correlation coefficients were used to present the relationship between the selected physicochemical parameters and sampling time. To show differences between the groups (defined according to the sampling spot), tests between independent groups were applied. Statistical tests were performed on standardized raw data after removing missing data.

The correlation between physicochemical parameters and sampling time was investigated with Spearman’s rank correlation coefficient *R*(Gauthier 2001), which is applicable for ordinal and quantitative variables. The *p* value threshold for statistical significance was *p* = 0.05. Spearman’s correlation coefficient ranges from −1 to 1, with a positive value representing a positive correlation — an increase or decrease in the value of both analyzed variables. When the values of one variable tend to decrease with an increase of the other one, then the correlation coefficient is negative. It is assumed that correlation is very weak when the absolute value of the coefficient is in the range from 0 to 0.3, fairly strong in the range from 0.3 to 0.5, and very strong when the coefficient value is close to 1. The correlation between sampling time and the following physicochemical parameters were investigated: pH, electrical conductivity, TOC, concentrations of ammonia (NH_4_), boron, barium, calcium, iron, potassium, lithium, magnesium, manganese, sodium, silica, strontium, chloride, and bromide. The calculated Spearman’s rank correlation coefficients are presented in Table 1.

**Table 1:**
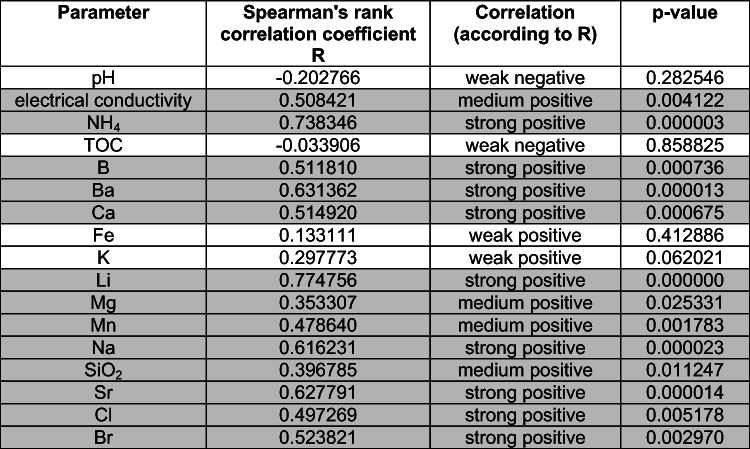
Results of R-Spearman correlation: day of sampling versus parameters values

In gray indicated *p* value less than 0.05, which means statistically significant

There is a statistically significant positive correlation between sampling time and the following parameters: electrical conductivity, concentration of ammonia, strontium, sodium, barium, chloride, bromide, calcium, boron, manganese, lithium, silica, and magnesium. The results suggest that concentrations of those chemicals increase with an increase of duration time of contact between fracturing fluid and reservoir rock. They were likely leached from the rock during hydraulic fracturing in high pressure and temperature conditions.

The concentration of potassium and iron and pH value and TOC are independent of sampling time; there is no statistically significant correlation between sampling time and those parameters.

Further, statistical analysis was devoted to changes of physicochemical parameters of flowback fluids between a well head and a wastewater storage tank. At the surface, the flowback fluid is equilibrated to ambient conditions. The pressure is reduced; after cooling down, the solid suspension is removed from flowback fluids, and gaseous and organic phases are removed in 2 or 3 phases separator. Finally, the flowback fluid is collected in a tank with free access to atmospheric air (regardless if tanks are closed or open, there is always air above the fluid surface). As a result, the physicochemical parameters of the flowback fluid change during the collection process.

The sampling time and spot are known for each sample of the flowback fluid in the analyzed dataset. Based on this, 4 groups were distinguished, from the unrepresentative to the most representative fluid samples. This classification was made on the basis of expert knowledge, the experience, and observations of people collecting samples.

Hence, the samples can be divided in four groups: (A) samples from initiation of recovery process, which composition is different than fluid received latter; mainly the solution used for drilling the plugs; samples may be unrepresentative in terms of the characteristics of the bulk flowback fluid for its further management; usually, small amounts of this kind of waste could be generated on the well pad; (B) samples taken before the first stage of treatment, without preliminary separation of gas and suspended solids; in normal operation such waste could be received only as a result of line failure or loss of separator efficiency; (C) samples of flowback fluid after the initial (on-site) treatment, taken from the reservoir tank; mixture of different batches of the pretreated fluid from fracturing process collected over several hours; (D) samples taken from a collective tank, different batches of the pretreated flowback fluid collected for a long time; the most representative for the bulk flowback — a mixture of pretreated flowback fluids from all stages of fracturing process.

**Fig. 6:**
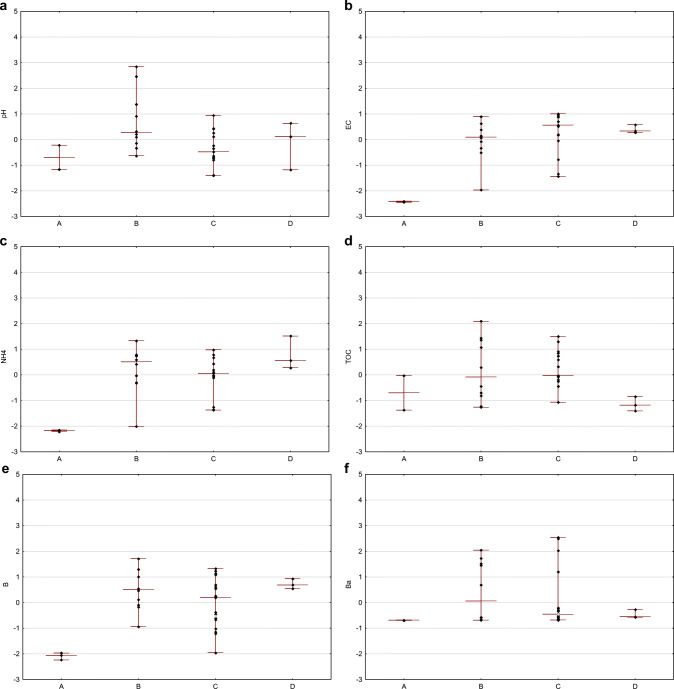

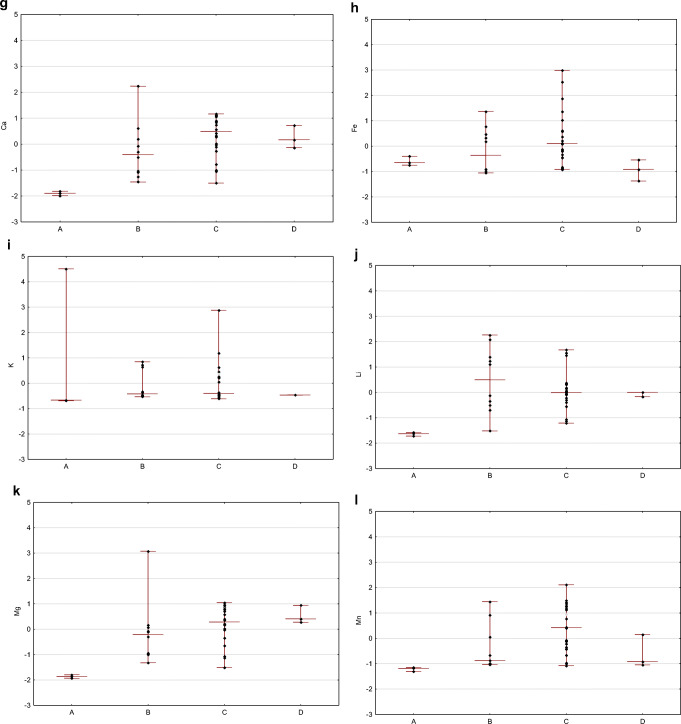

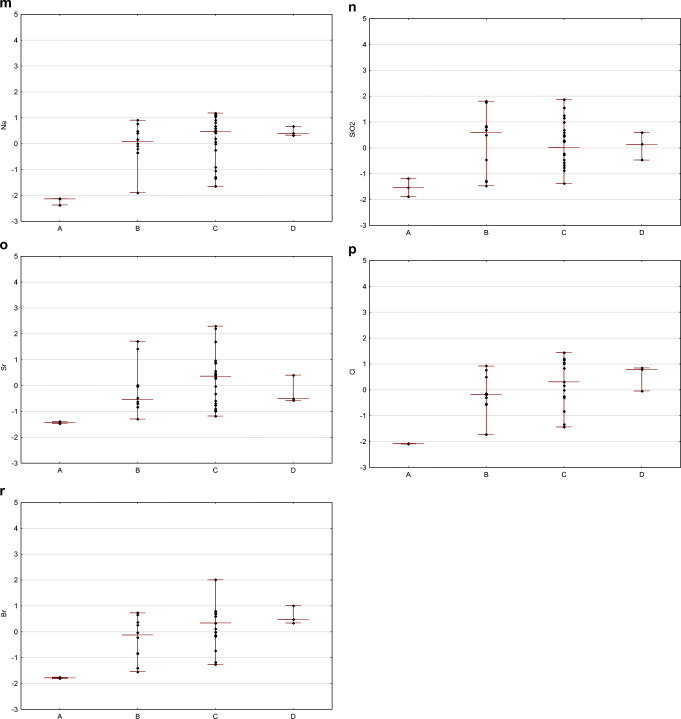
Group-related differentiation of flowback: standardized values of pH (**a**), electric conductivity (**b**), ammonia (**c**), total organic carbon (**d**), boron (**e**), barium (**f**), calcium (**g**), iron (**h**), potassium (**i**), lithium (**j**), magnesium (**k**), manganese (**l**), sodium (**m**), silica (**n**), strontium (**o**), chlorides (**p**), and bromides (**r**) are given (diamonds) in relation to the level of representativeness (*x*-axis) (groups are defined in the text), within the groups maximum and minimum is marked with short line and median is marked with long line

The differentiation between the groups is presented by the concentration ranges and the medians in each group in Fig. 6 (standardized data used).

The correlation between groups was investigated with the nonparametric Kruskal–Wallis test (Kruskal 1952). The grouping variable was ordinal, from the least (group A) to the most representative (group D). Quantitative variables were standardized. The null hypothesis of no difference between the groups was tested. The *p* value threshold for statistical significance was *p* = 0.05. The results of the test are presented in Table 2.

**Table 2:**
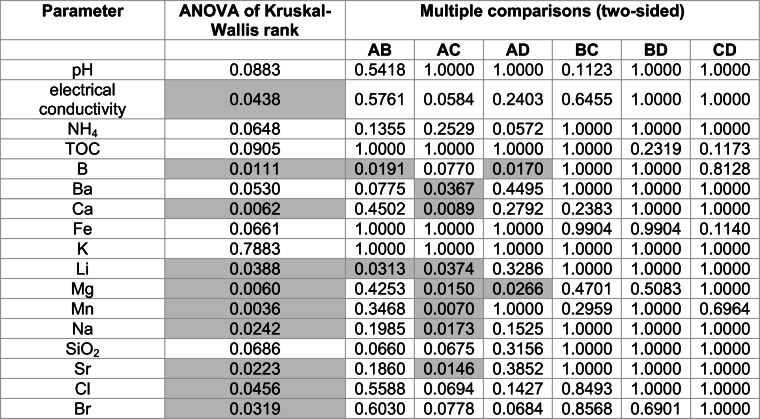
Results of ANOVA of Kruskal–Wallis rank and post hoc multiple comparisons (two sided); representativeness as a grouping variable

In gray indicated *p* value less than 0.05, which means statistically significant

A statistically significant result of the Kruskal–Wallis test, meaning that at least one group differs from the other one, was obtained for the following parameters: electrical conductivity, concentration of boron, calcium, lithium, magnesium, manganese, sodium, strontium, chloride, and bromide.

Post hoc tests were used in order to indicate the group which differed the most from other groups (Siegel and Castellan Jr. 1988). For this purpose, multiple comparisons of mean ranks for all statistics were used. It was found that the flowback samples in group A differed from groups B and D by concentration of boron; from group C by concentration of barium, manganese, sodium, and strontium; and from groups C and D by magnesium. The samples from group A may be used in the study on the interaction between fracturing fluid and reservoir rock; however, they cannot be used in the study on the development of the treatment process of flowback fluid. The composition of samples from group A varied the most from other groups and did not represent the average composition of waste produced after the fracturing process. The differences in the composition of samples from groups B, C, and D were not significant.

The applied statistical tests confirmed a significant relationship between the properties of flowback fluid (expressed by the value of a given parameter) and the time of sampling and sampling spot. The following parameters differentiated the most: electrical conductivity, concentration of NH_4_, boron, barium, calcium, lithium, magnesium, manganese, sodium, silicon, strontium, chlorides, and bromides. Otherwise, for pH, TOC, concentration of potassium, and iron, there were no changes observed due to sampling time or sampling spot.

### Determining the ranges of parameters characteristic for Polish flowback fluids

Knowing the chemical constituents of flowback is crucial for informative decisions on its appropriate management. Based on this information, it can be concluded whether the collected fluid can be recovered or neutralized (including neutralization by disposal). In addition, this information should be also used to prepare a mining waste management program, which, in accordance with legal regulations, is obligatory to enable the waste to be deposited in the mining waste treatment facility. The data on the chemical composition of flowback fluids available in the literature most often provided the ranges of selected parameters. Such generalized information is sufficient for preliminary works, as long as the user makes an independent assessment of the credibility and reliability of the processed data. Even assuming that the results met all the standards for analytical uncertainty, the reliability of descriptive statistics may also be affected by selection and number of samples, place and method of sampling, and sampling time, whether the samples came from one or several boreholes or/and treatments or are they characteristic for specific geographic location.

Within the analyzed dataset, 3 out of 40 samples were considered unrepresentative, based on the detailed expertise on the technological process of fracturing and the circumstances of the samples collection (time and place of sampling), as well as on the basis of statistical testing results (samples from group A differed from others).

In Table 3, the ranges, arithmetic averages, and medians are provided for selected parameters: TDS, concentration of chloride, sodium, calcium, potassium, barium, bromide, iron, strontium, and lithium. The presented characteristics were first calculated for the full dataset (40 samples), then taking into account only representative samples (37 samples).

**Table 3:** Comparison of descriptive statistic of full dataset versus representative dataset

1	2				3				4			
Parameter	Characteristics of the complete set (40 samples)				Characteristics of the a representative set (37 samples)				Change in characteristics			
	Min	Max	Mean	Median	Min	Max	Mean	Median	Min	Max	Mean	Median
TDS	20,182	123,815	82,270	77,424	20,182	123,815	82,270	77,424	0	0	0	0
Cl	4100	79,000	48,440	47,750	12,000	79,000	51,593	50,000	7900	0	3153	2250
Na	215	28,750	19,232	22,229	4017	28,750	20,670	22,439	3802	0	1438	210
Ca	62	11,712	5543	6036	1400	11,712	5968	6308	1338	0	425	272
K	82	10,240	1422	529	228	7040	1255	536	146	−3200	−167	8
Ba	0.2	1710	368.0	114.8	5.4	1710	397.4	127.2	5.2	0	29.4	12.4
Br	25.0	1100	533.9	585.0	100.0	1100	569.7	617.5	75	0	35.8	32.5
Fe	0.5	105.0	33.1	28.5	0.5	105.0	34.2	30.0	0	0	1.2	1.5
Sr	0.2	2230	870.1	851.5	106.0	2230	938.7	857.0	105.9	0	68.6	5.5
Li	0.1	28.9	12.5	12.2	1.5	28.9	13.5	12.5	1.4	0	1.0	0.3

Table 3, column 4, shows the differences in indicators between the presented datasets. Discarding unrepresentative samples eliminates the extreme potassium concentration found in one of the samples. The mean and median values for the representative set to change by a few percent in relation to the full set. In the case of TDS, no changes were made, because (coincidentally) in three samples considered unrepresentative, this parameter was not determined. The inclusion of unrepresentative samples underestimates the minimum performance levels. The minima of both sets do not differ in the case of iron concentration, while in the case of other parameters, the differences are significant: for chloride and potassium concentrations, the minimum values are three times higher, and for bromide concentrations, four times higher; for sodium, calcium, barium, and lithium, the difference is at least an order of magnitude, and for strontium, it is almost 3 orders of magnitude.

### Comparison of the chemical composition of flowback fluids from Poland and worldwide localizations

**Fig. 7:**
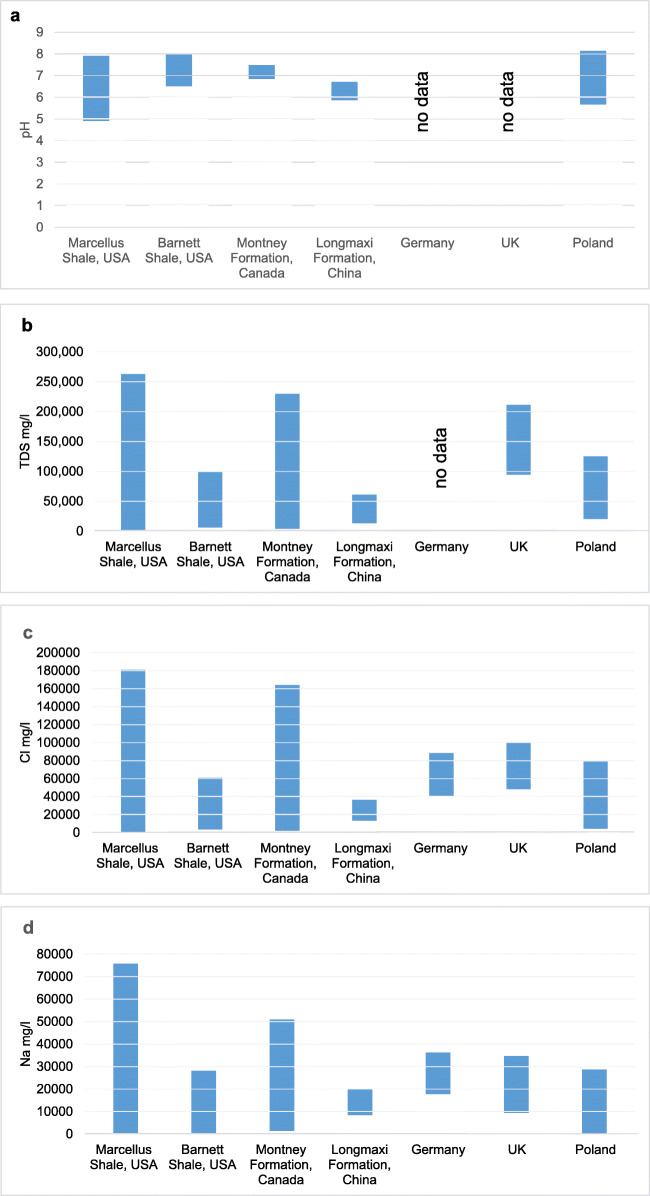

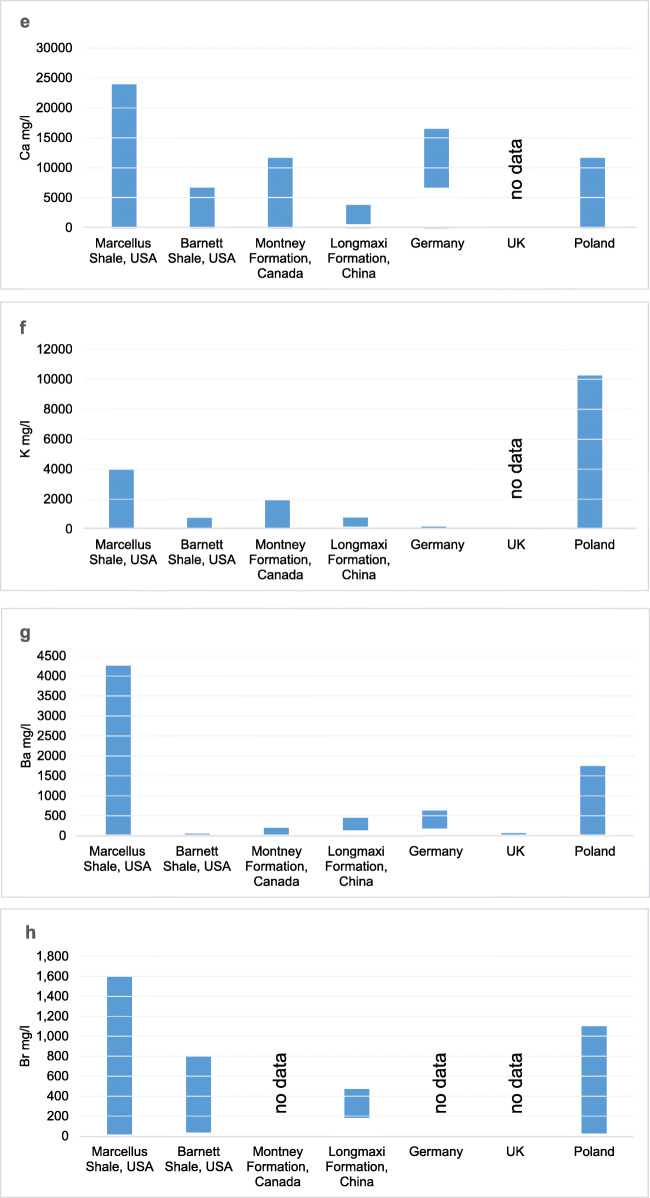

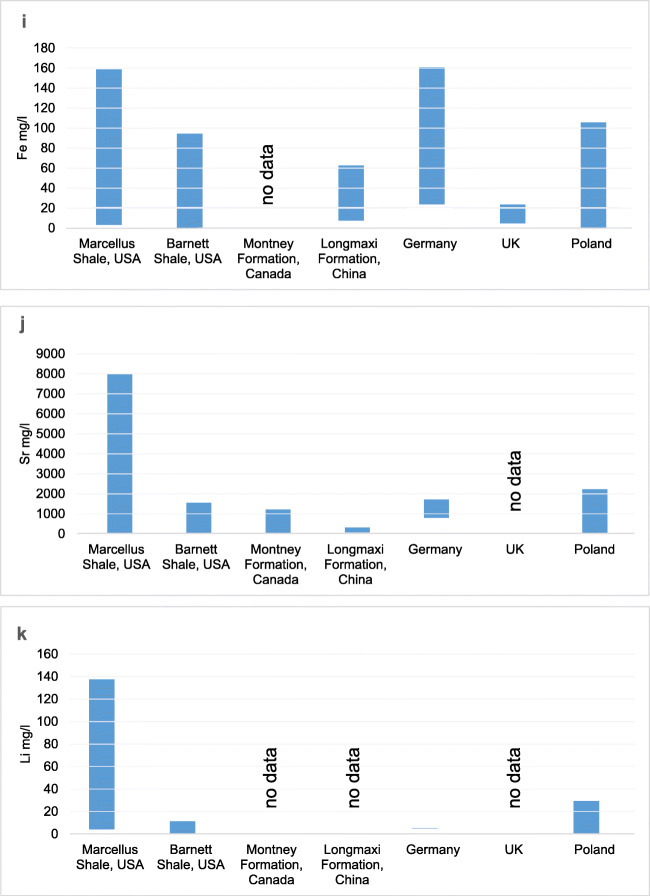
**a**–**k** Comparison of flowback characteristics in different regions

According to the data presented, flowback fluids from different regions of the world vary a lot in their chemical composition (a large range of values for individual analyzed parameters). The differences and similarities of ranges of physicochemical parameters of flowback fluids are shown in Table 4 and presented in graphs (Fig. 7).

**Table 4:** Comparison of chemical characteristics of flowback fluid from different regions of the world

	**USA**	**USA**	**Canada**	**China**	**China**	**Germany**	**UK**	**Poland**
**Parameter**	**Marcellus**	**Barnett**		**Longmaxi**	**Tarim Basin**			
pH	4.9–7.9	6.5–8.0	2.3–9.5	6.84–7.48	5.86–6.71	nr	nr	5.66–8.14
TDS	680–261,000	5850–97,000	3609–228,259	13,000–60,000	nr	nr	94,000–210,000	20,182–123,815
Cl	64.2–181,000	3300–60,800	1893–164,018	13,110–36,470	32,000–64,000	40,360–88,440	48,000–100,000	12,000–79,000
Na	63.8–73,800	278–28,200	1344–51,027	8296–19,830	nr	17,690–36,390	9300–34,800	4017–28,750
Ca	35.2–24,000	13–6730	13–11,705	564–3870	nr	6700–16,550	nr	1400–11,712
K	2.69–3950	4–750	49–1920	157–768	nr	52–157	28.8–40.6	228–7040
Ba	0.33–4220	0.05–17.9	<1–467	138–412	nr	180–593	9.20–30.0	5–1710
Br	15.8–1600	34.3–798	2.1–38	180–470	nr	nr	nr	100–1100
Fe	2.68–158	nd–93.80	nr	7–62	nr	23–160	4.20–23	<1–105
Sr	0.58–8020	nd–1550	3.7–1263	67–320	nr	790–1720	nr	106–2230
Li	4.06–137	0.05–10.80	<1–49	nr	nr	5–6	nr	1.46–28.9

*nd*, not detected; *nr*, not reported

Except for flowback fluids from Canada, the pH values of the fluids were close to neutral. Canadian flowback’s pH represented a wide range from acidic to alkaline. The highest concentration of TDS above 200,000 mg/l was observed in flowback fluids from the USA (Marcellus), Canada, and the UK. The concentration of TDS in the fluids from China was almost four times lower compared to those mentioned earlier.

All fluids were characterized by high salinity, though their values differed significantly. Similar ranges were observed in European fluids (Germany and the UK); however, in the case of Poland, the minimum observed value was four times lower than that for fluids from other locations.

In all fluids, the high concentration of calcium and sodium was observed. The highest concentration of sodium was found in fluids from the Marcellus deposit in the USA and Canada, whereas the lowest concentration was found in the fluids from Poland, China, and Barnett deposit in the USA. The concentration of calcium was the highest in the fluids from the Marcellus deposit in the USA and from Germany. The lowest concentration of calcium was observed in flowback fluids from China. In Poland and Canada, calcium concentrations were very similar.

The concentration of potassium was lower than sodium and calcium, and its highest values were observed in Poland. The highest concentration of barium was determined in fluids from the Marcellus deposit in the USA, whereas the lowest in fluids from Barnett deposit also in the USA and in the UK. The lowest concentration of bromide was observed in Canadian flowback fluids.

The highest concentration of iron was observed in flowback fluids from the Marcellus deposit and from Germany. In Poland and China, iron was present at lower level, being the lowest in flowback analyzed in the UK.

Strontium and lithium were most abundant in flowback fluids from the Marcellus deposit. The lowest concentration of lithium was detected in samples from Germany.

The values of other tested parameters were generally diverse; however, for individual sedimentation basins, higher values of some parameters were identified as a specific feature of the region. It is important to note that the concentration of sulfate was low in all tested flowback fluids. In the Polish flowback fluids in almost 90% of samples, this parameter was not detected above the LoQ of the testing method.

### The possibilities of flowback fluids management

The variability of the chemical composition of flowback fluids influences the proper way of their management.

The principles of management of flowback fluids are defined by the European Union law — the Directive on the management of waste from extractive industries and also the Directive of waste (in the scope not covered by the Directive on the management of waste from extractive industries). According to this law, the flowback fluids are classified as mining waste produced during ore and other minerals processing and belong to a group named *other not listed waste* with code number 01 05 99. The use of methods aimed at preventing the generation of extractive waste is required. If it is not possible to prevent waste generation, the mining waste should be recovered and eventually disposed of (including depositing in a waste facility).

Flowback fluid should be preferably reused. Otherwise, it is directed to a treatment plant (including wastewater treatment plant) or a waste facility (Directive 2006/21/EC of the European Parliament and of the Council of 15 March 2006).

In terms of technology, there are several methods for flowback fluids treatment reported in the literature (Olsson et al. 2013; Konieczyńska et al. 2016-unpublished; Butkovskyi et al. 2018; Sun et al. 2019). The preliminary treatment methods frequently used for the removal of suspended solids and heavy metals are sedimentation, filtration, flotation, and coagulation. Oxidation and adsorption on granulated activated carbon are used for the removal of organic substances. Removal of dissolved solid is usually performed by evaporation and crystallization and also with membrane processes such as reverse osmosis and nanofiltration (Olsson et al. 2013). Ion exchange processes may be also applied for the removal of dissolved salt. During treatment processes, some valuable elements could be recovered from flowback fluids (Konieczyńska et al. 2016 unpublished).

In order to sustain the effectiveness of any recovery process of the flowback fluid, its chemical composition stability over time should be controlled based on selected indicators. In case of flowback fluids from Poland, the treatment processes have to be tailored to the following concentration ranges: TDS from 20 to 124 g/l, Cl^−^ from 12 to 80 g/l, Na^+^ from 4 to 30 g/l, Ca^2+^ from 1 to 12 g/l, K^+^ from 200 to 7500 mg/l, Ba^2+^ from 5 to 1800 mg/l, Br^−^ from 100 to 1100 mg/l, Fe^2+,3+^ from 0 to 105 mg/l, Sr^+^ from 100 to 2500 mg/l, and Li^+^ from 0 to 30 mg/l.

The methods of flowback fluids treatment in Poland complies with the requirements outlined in the Directive on management of waste from extractive industries and with the Polish legal framework. Flowback fluids can be recovered or disposed of (in an installation) or stored (in a mining waste facility). The appropriate permits and decisions on the processing and storage are required for both installation and facility. Also, the transferred fluids should meet the requirements in terms of compliance of their chemical composition with the chemical composition presented in these permits and decisions. Additionally, a person providing flowback fluids (the holder and/or producer of waste) should have a mining waste management program, which indicates that this waste may be recovered (or stored) by processes addressed in the permits. The underground storage of flowback is possible under the procedure of permitting and is limited to other than hazardous and inert mining waste.

The appropriate management of large volumes of liquid waste reduces the risk for people and for environment losses.

## Conclusions

The flowback fluids are characterized by high salinity, caused mainly by high concentrations of chloride, sodium, calcium, and potassium. The high concentrations of barium, bromide, strontium, lithium, and iron were also found in flowback fluids. The composition of flowback fluids varied between geographical locations and also because of different technologies used for hydraulic fracturing and completion treatments.

In case of flowback fluids from Poland, they are characterized by TDS in the range from 20,182 to 123,815 mg/l, chloride from 12,000 to 79,000 mg/l, sodium from 4017 to 28,750 mg/l, calcium 1400 to 11,712 mg/l, potassium from 228 to 7040 mg/l, barium from 5.4 to 1710 mg/l, bromide from 100 to 1100 mg/l, iron from 0.5 to 105 mg/l, strontium 106 to 2230 mg/l, and lithium from 1.5 to 28.9 mg/l.

Statistical analyses revealed a relation between sampling schedule and the following parameters: electrical conductivity concentration of ammonia, boron, barium, calcium, lithium, magnesium, manganese, sodium, strontium, chloride, bromide, and silica. On the contrary, the pH and concentration of potassium and iron were irrespective of the sampling schedule (meaning time and spot of sample collection).

The diversity of the characteristics of flowback fluids is the main factor that determines the selection of a waste management method. Due to the presented variability of the chemical composition of flowback over time, it is recommended to monitor the composition by continuous measurements of selected indicators during the whole treatment process. This contributes to proper and safe handling especially in the case of exploration wells in new regions. Such monitoring ensures the technical safety of the installation neutralizing this type of waste and prevent from exceeding any threshold values of the parameters permitted for a given treatment measure.

In the case of waste characterized by high variability of chemical composition over time, the general rule for determining properties before the management should include the appropriate selection of the sampling site so that the obtained results present unified average parameters readings and not random “peaks or valleys” of values.

## Data Availability

The datasets used and/or analyzed during the current study are available from the authors on reasonable request.
